# Complete Genome Sequence of *Alternanthera mosaic virus*, Isolated from *Achyranthes bidentata* in Asia

**DOI:** 10.1128/genomeA.00020-16

**Published:** 2016-03-17

**Authors:** Nozomu Iwabuchi, Tetsuya Yoshida, Akira Yusa, Shuko Nishida, Kazuyuki Tanno, Takuya Keima, Takamichi Nijo, Yasuyuki Yamaji, Shigetou Namba

**Affiliations:** Department of Agricultural and Environmental Biology, Graduate School of Agricultural and Life Sciences, The University of Tokyo, Tokyo, Japan

## Abstract

*Alternanthera mosaic virus* (AltMV) infecting *Achyranthes bidentata* was first detected in Asia, and the complete genome sequence (6,604 nucleotides) was determined. Sequence identity analysis and phylogenetic analysis confirmed that this isolate is the most phylogenetically distant AltMV isolate worldwide.

## GENOME ANNOUNCEMENT

*Alternanthera mosaic virus* (AltMV) is a member of the genus *Potexvirus* in the family *Alphaflexiviridae* and has flexuous filamentous particles and a single-stranded positive-sense RNA genome. The AltMV genome is about 6.6 kb in size and contains five open reading frames (ORFs). AltMV was first described from *Alternanthera pungens* (family *Amaranthaceae*) in Australia (1) and then from plants in at least eight families in the United States, Europe, and Brazil (2). Here, we present the first complete genome sequence of the Asian isolate of AltMV.

*Achyranthes bidentata* (family *Amaranthaceae*) is a perennial herb distributed widely in Asia. In 2015, *A. bidentata* leaves showing mosaic symptoms were collected in Tokyo, Japan. The presence of potexvirus-like particles with lengths of 500 to 600 nm was confirmed by electron microscopy. Virion purification and viral RNA extraction were performed, as described previously (3). cDNA was synthesized from the extracted RNA using avian myeloblastosis virus reverse transcriptase (Nippon Gene, Japan) with an oligo(dT) primer. An approximately 800-bp fragment was amplified by PCR with primers specific for potexvirus ORF1 (4), cloned to pCR-Blunt II TOPO vector (Invitrogen, USA), and sequenced with vector-specific primers. Using a BLASTn search against the GenBank database, the obtained sequence showed 82% sequence identity to the partial sequences of ORF1 of known AltMV isolates. Overlapping fragments were amplified with primers designed from the obtained sequence and known AltMV complete genome sequences (5–7), and oligo(dT). The 5′ end of the genome was amplified using the Rapid Amplification of cDNA Ends (RACE) system (Invitrogen, USA). Each fragment was cloned into pCR-Blunt II TOPO vector and sequenced. The complete genome sequence, 6,604 nucleotides excluding the poly(A) tail at its 3′ end, was reconstructed by assembling all overlapping sequences, which displayed 100% identity in the overlapping regions. We first detected AltMV in Asia and from a plant in the genus *Achyranthes*, and designated this *A. bidentata*-infecting isolate as AltMV-Ac.

The sequence identities between AltMV-Ac and other AltMV isolates were calculated using the program SDT (8) based on pairwise alignments using the MUSCLE algorithm. The identities of ORF1 to ORF5 were 78.7 to 79.5%, 77.5 to 78.5%, 77.8 to 79.0%, 71.4 to 74.0%, and 78.5 to 80.0%, respectively, at the nucleotide levels; and were 88.8 to 89.7%, 84.1 to 85.8%, 85.5 to 87.3%, 63.5 to 66.7%, and 90.3 to 92.8%, respectively, at the amino acid levels. The 5′- and 3′-untranslated regions of AltMV-Ac showed 89.4 to 91.5% and 90.0 to 93.5% identities, respectively. The amino acid sequences of ORF5 (coat protein) were aligned using the MUSCLE algorithm, and a phylogenetic analysis was performed in MEGA version 6.06 with the neighbor-joining algorithm using 1,000 replicates for bootstrapping. Phylogenetic analysis revealed that AltMV-Ac belonged to a clade with other AltMV isolates within the genus *Potexvirus*. Interestingly, within this clade, AltMV-Ac branched first and the other 17 isolates formed a monophyletic group (93.2 to 100% identity), as previously described (2). Taken together, AltMV-Ac is the phylogenetically most distant AltMV isolate worldwide.

### Nucleotide sequence accession number.

The genome sequence of AltMV-Ac has been deposited into DDBJ under accession number LC107515.
